# Reward and Behavioral Factors Contributing to the Tonic Activity of Monkey Pedunculopontine Tegmental Nucleus Neurons during Saccade Tasks

**DOI:** 10.3389/fnsys.2016.00094

**Published:** 2016-11-11

**Authors:** Ken-ichi Okada, Yasushi Kobayashi

**Affiliations:** ^1^Laboratories for Neuroscience, Visual Neuroscience Group, Graduate School of Frontier Biosciences, Osaka UniversityOsaka, Japan; ^2^Center for Information and Neural Networks, National Institute of Information and Communications Technology, Osaka UniversityOsaka, Japan; ^3^Research Center for Behavioral Economics, Osaka UniversityOsaka, Japan

**Keywords:** reward, saccade, reinforcement learning, Parkinson’s disease, movement execution

## Abstract

The pedunculopontine tegmental nucleus (PPTg) in the brainstem plays a role in controlling reinforcement learning and executing conditioned behavior. We previously examined the activity of PPTg neurons in monkeys during a reward-conditioned, visually guided saccade task, and reported that a population of these neurons exhibited tonic responses throughout the task period. These tonic responses might depend on prediction of the upcoming reward, successful execution of the task, or both. Here, we sought to further distinguish these factors and to investigate how each contributes to the tonic neuronal activity of the PPTg. In our *normal* visually guided saccade task, the monkey initially fixated on the central fixation target (FT), then made saccades to the peripheral saccade target and received a juice reward after the saccade target disappeared. Most of the tonic activity terminated shortly after the reward delivery, when the monkey broke fixation. To distinguish between reward and behavioral epochs, we then changed the task sequence for a block of trials, such that the saccade target remained visible after the reward delivery. Under these *visible* conditions, the monkeys tended to continue fixating on the saccade target even after the reward delivery. Therefore, the prediction of the upcoming reward and the end of an individual trial were separated in time. Regardless of the task conditions, half of the tonically active PPTg neurons terminated their activity around the time of the reward delivery, consistent with the view that PPTg neurons might send reward prediction signals until the time of reward delivery, which is essential for computing reward prediction error in reinforcement learning. On the other hand, the other half of the tonically active PPTg neurons changed their activity dependent on the task condition. In the normal condition, the tonic responses terminated around the time of the reward delivery, while in the visible condition, the activity continued until the disappearance of the saccade target (ST) after reward delivery. Thus, for these neurons, the tonic activity might be related to maintaining attention to complete fixation behavior. These results suggest that, in addition to the reward value information, some PPTg neurons might contribute to the execution of conditioned task behavior.

## Introduction

Humans and other animals select and execute appropriate behavior moment by moment, based on the prediction of the upcoming reward. In this context, when we obtain or loose a reward, we must acquire and renew our behavioral policy. In such learning situations, the dopaminergic neurons in the midbrain substantia nigra pars compacta (SNc) and ventral tegmental area (VTA) are thought to play a pivotal role by encoding a reward prediction error signal (Schultz, 2002; Bromberg-Martin et al., 2010). The pedunculopontine tegmental nucleus (PPTg, also known as PPTN or PPN) in the brainstem is one structure that projects to these dopaminergic neurons, and regulates the firing of dopaminergic neurons (Mena-Segovia et al., 2008). Neurons in the PPTg are involved in reward processing and learning. Lesions of the PPTg in rats blocked the positive reinforcing effects of morphine and amphetamine (Bechara and van der Kooy, 1989) and disrupted learning (Dellu et al., 1991), and PPTg neuronal responses to reward were reported in various species, including rats (Norton et al., 2011), mice (Thompson and Felsen, 2013), cats (Dormont et al., 1998) and monkeys (Kobayashi et al., 2002; Okada et al., 2009; Hong and Hikosaka, 2014). We previously reported that a population of PPTg neurons exhibited tonic responses throughout the task period of a conditioning task, and some of them showed a significant dependency on the magnitude of the predicted reward (Okada et al., 2009). This property of the signal matches that of the reward prediction signal that is necessary for the computation of reward prediction error as represented by dopaminergic neurons.

While the PPTg preferentially projects to the dopaminergic neurons of the SNc (Watabe-Uchida et al., 2012), other studies suggested that neurons in the PPTg also carry motor and reward signals to the dopaminergic neurons of the VTA (Dautan et al., 2016). The PPTg also connects with various limbic and motor structures (Nakano, 2000; Mena-Segovia et al., 2004), and is postulated to be an integral component of the limbic-motor interface (Winn et al., 1997). Single PPTg neurons respond to various modalities of task events, including sensory, motor, and reward (Dormont et al., 1998; Kobayashi et al., 2002; Thompson and Felsen, 2013). The classical literature regarded the PPTg as a locomotor center (Garcia-Rill and Skinner, 1988), but more recent studies suggest that the PPTg is related to the execution of conditioned behavior. Dysfunction of the PPTg did not disrupt locomotion or feeding behavior, but impaired performance on several conditioned task behaviors (Inglis et al., 1994; Condé et al., 1998; MacLaren et al., 2014). We previously reported conditioned behavior-related activity in PPTg neurons, as some PPTg neurons showed activity modulation only for conditioned task saccades and not for spontaneous saccades outside the task (Okada and Kobayashi, 2009). The tonic activity of PPTg neurons also related to the behavioral performance of monkeys (Kobayashi et al., 2002; Okada et al., 2009), and it started prior to the appearance of the initial stimulus and were related to the anticipatory fixation behavior (Okada and Kobayashi, 2013). The tonic activity of PPTg neurons might play a role in executing conditioned task behavior by maintaining the motivational and/or attentional state of the animal (Steriade, 1996).

While the tonic activity of PPTg neurons was related to prediction of upcoming reward and execution of fixation behavior, the functional relationship between those activities remains unclear. Here, we sought to further distinguish these factors and to investigate how each factor contributes to the activity of neurons in the PPTg. To clarify the functional significance of tonic activity for behavior and learning, we examined individual, tonically active PPTg neurons during two task conditions that distinguished between reward and behavioral epochs.

## Materials and Methods

### General

We recorded the neuronal activity of neurons in the PPTg in three Japanese monkeys (*Macaca fuscata*; monkey 1 and 2, male; monkey 3, female) while they performed a visually guided saccade task. All experimental procedures were approved by the Committee for Animal Experiments at Okazaki National Research Institutes and Osaka University and are in accordance with the National Institutes of Health *Guidelines for the Care and Use of Laboratory Animals*.

Information on the experimental procedures was published previously (Kobayashi et al., 2002). In short, a head holding device, a recording chamber, and a scleral search coil were implanted under general anesthesia. The position of the recording chamber was determined based on MRI data (2.2 T; Hitachi; Okada et al., 2009). The task was controlled and behavioral and neuronal data were stored in a personal computer-based, real-time data acquisition system (TEMPO; Reflective Computing). Eye position was sampled using a search coil method at a spatial resolution of 0.1° and time resolution of 1 ms. Neuronal activity of single neurons was recorded with tungsten microelectrodes (impedance of 1–6 MΩ, FHC) and was isolated by the shape of action potentials using a template matching algorithm (MSD; Alpha Omega).

### Behavioral Task

During the experiment, the monkeys were seated in a primate chair and placed in front of the screen of a 21″ cathode ray tube monitor in a dark, sound-attenuated room. The monkeys performed a visually guided saccade task, during which they made saccades to a peripheral visual target for a juice reward. Initially, the fixation target (FT, a circle of 0.8°) appeared at the center of the screen and the monkey had to fixate on it within 3000 ms to a precision of ±2° and maintain the fixation for a variable duration (400–1500 ms). Then, a saccade target (ST, a circle of 0.8°) appeared at an eccentricity of 10° from the FT in 1 of 2 (left or right) or 8 (0, 45, 90, 135, 180, 225, 270, and 315°) possible directions. Monkeys were required to saccade to the ST within 80–500 ms to a precision of ±2°. The trials that the monkeys maintained fixation on the ST for 300 ms were regarded as successful. If the monkey broke fixation at any time during the fixation period, failed to make a saccade to the ST, or broke fixation to the ST, an error tone sounded and the trial was aborted. In the *normal* saccade task, the ST disappeared 400 ms after the saccade (ST remained visible 100 ms after performance inspection), and after a 400-ms delay, rewards (some drops of juice) were presented together with a tone. The next trial started after an intertrial interval of 1.5–2 s.

**Figure 1:**
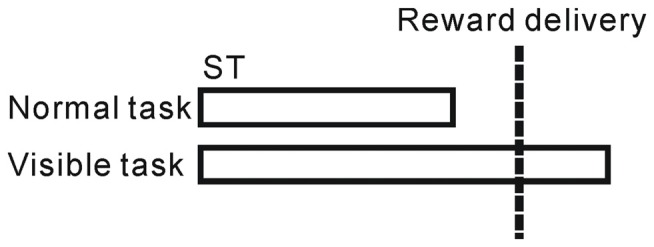
**Schematic illustration of the normal and visible tasks.** In the normal task, the saccade target (ST) disappeared before the reward delivery, while in the visible task, the ST remained visible even after the reward delivery.

To distinguish between reward and behavioral epochs, we changed the above task sequence for a block of trials (20–30 trials) with no apparent cue. In the *normal* task condition described above, ST disappeared before the reward delivery, while in the *visible* task condition, the ST remained visible even after the reward delivery (until 500 ms after the reward, Figure 1). Under these visible task conditions, the monkeys tended to maintain fixation on the ST even after the reward (see “Results” Section). Thus, the two task sequences enabled us to separate the prediction of upcoming reward and execution of fixational behavior in time.

### Data Analysis

Our database consisted of 296 neurons (216, 27, and 53 neurons for monkeys 1–3, respectively). These 296 neurons all showed tonic increases in activity during the normal visually guided saccade task, as we previously reported (Okada and Kobayashi, 2013). Within this sample of neurons, 148 were recorded from under both normal and visible task conditions.

To analyze and display neuronal activity around the time of reward, the normalized activity of each neuron was calculated as a receiver operating characteristic (ROC) value, comparing the firing rate of the neuron collected in a 200-ms window centered on that time vs. the firing rate collected during a post-reward period represented by a 500–1000 ms after the reward delivery. Termination of the tonic activity was estimated using a method based on the cumulative sum of the ROC values (Falzett et al., 1985).

For the analysis of monkey behavior during the normal and visible tasks, we extracted the fixation break behavior when they stopped watching the ST (i.e., when the gaze exited a 3° window around the ST). A fixation break <0 ms implies that the monkey broke fixation before reward delivery, while a fixation break >0 ms implies that the monkey maintained fixation even after reward delivery.

Because there were normal/visible task-related differences in fixation behavior during the reward period (0–600 ms after the onset of the reward, see “Results” Section), task-related changes in neuronal activity were defined based on their significant increase, decrease, or no significant change in activity during that reward period for the normal vs. visible task conditions (*p* < 0.05, Wilcoxon rank-sum test). To examine the changes in the neuronal activity after the task change, we compared the firing rates during the reward period for the five trials before and after task change (Scheffé test, *p* < 0.05), and compared the response in the first trial after task change with the previous five trials (Scheffé test, *p* < 0.05). We defined the normal/visible task-related modulation as the ROC value comparing the firing rate in the reward period between normal vs. visible trials. For the analysis of anticipatory behavior-related modulation, we used the reaction time to fixate on the FT (RTFT). We calculated behavior-related modulation by comparing the firing rate in the short vs. long RTFT trials for the pre-fixation period (0–600 ms before the appearance of the FT) using ROC analysis. The activity correlations with the initial fixation behavior-related modulation and normal/visible task-related modulation were assessed using Spearman’s rank correlation (Okada and Kobayashi, 2013).

## Results

**Figure 2:**
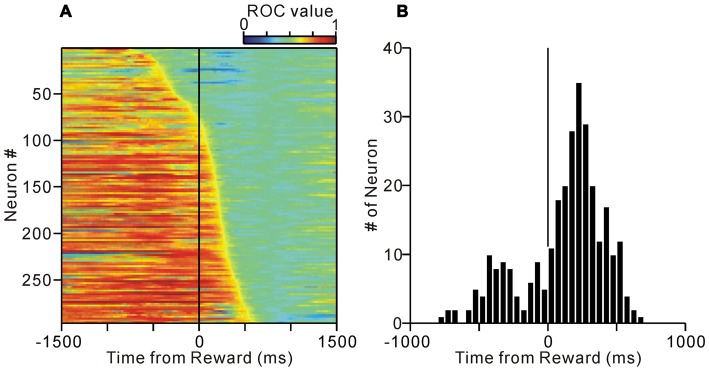
**(A)** Activity of the tonic excitatory neurons of the PPTg during the normal saccade task. The activity of each of the 296 PPTg neuron is presented as a row of pixels. The data are aligned to the time of reward delivery. The neurons were sorted in the order of their termination of tonic firing. The color of each pixel indicates the receiver operating characteristic (ROC) value based on a comparison of the firing rate during a post-reward period (500–1000 ms after the reward delivery) and a test window centered on the pixel (200-ms duration). The warm colors (ROC value >0.5) indicate higher firing rate relative to the post-reward period, whereas the cool colors (ROC value <0.5) indicate lower firing rate. **(B)** Histograms for the termination of tonic activity.

We previously examined the neuronal activity of the PPTg during a reward conditioned, visually guided saccade task, and reported that a population of PPTg neurons showed tonic responses throughout the task period (Okada et al., 2009). Some tonic changes in activity started prior to the appearance of the initial stimulus and were related to the anticipatory fixation behavior (Okada and Kobayashi, 2013). Here, we first examined the termination timing of tonic increases in activity during the normal task condition (Figure 1). Many tonic increases in activity terminated shortly after the reward delivery (peaked around 200 ms after reward), while the tonic activity of other neurons terminated shortly before the reward (Figure 2).

**Figure 3:**
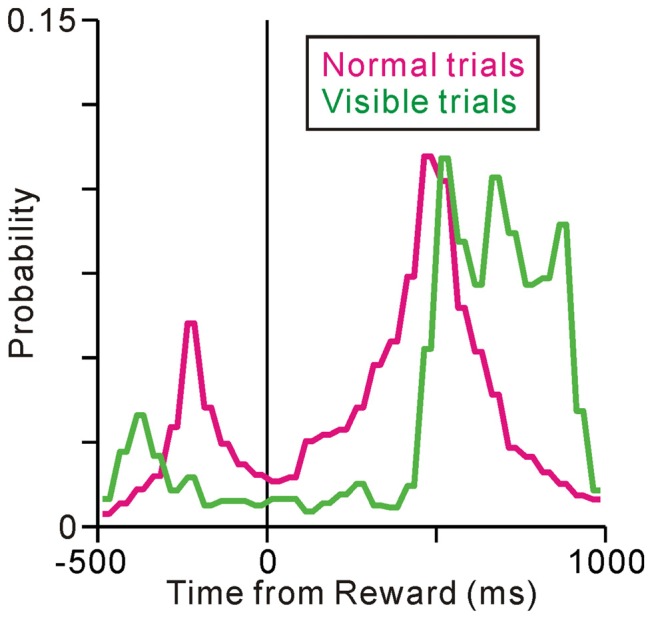
**Histograms for the fixation break times under the normal (magenta) and visible (green) task conditions**.

To examine whether the termination of tonic activity was related to the prediction of reward or execution of fixation behavior, we analyzed the monkeys’ fixation breaking behavior in successful trials. In the normal saccade task, there were basically two types of fixation breaks; in one (peak at about −200 ms) the monkeys broke fixation shortly after the disappearance of the ST but before the reward delivery possibly in response to the disappearance of the ST, in the other (peak at 500 ms) the monkeys broke fixation shortly after the reward delivery (Figure 3, normal condition). Thus, in the normal task condition, the end of an individual trial occurred almost at the same time as the delivery of reward and the end of fixation. Therefore, we could not distinguish neuronal activity related to prediction of upcoming reward from that associated with successful execution of the task behavior.

To distinguish between reward and behavioral factors in time, we changed the task sequence for a block of trials, such that the ST remained visible after the reward delivery (visible task, Figure 1). Note that, we did not change the performance inspection criteria; the monkeys were not required to fixate on the ST after reward delivery (rewards were already given). However, in the visible trials, the monkeys tended to maintain fixation even after reward delivery (Figure 3, visible condition). Comparing the normal task condition, one peak before the reward delivery is diminished possibly because there is no visual cue, while the other peak after the reward delivery is shifted later in time, yielding significantly longer fixation durations than under the normal task condition (*p* < 0.001, Wilcoxon rank-sum test). Therefore, in the visible task condition, the prediction of the reward and the end of an individual trial were separated in time. We recorded the activities of 67 neurons during both normal and visible task conditions and compared their activities under the two conditions.

**Figure 4:**
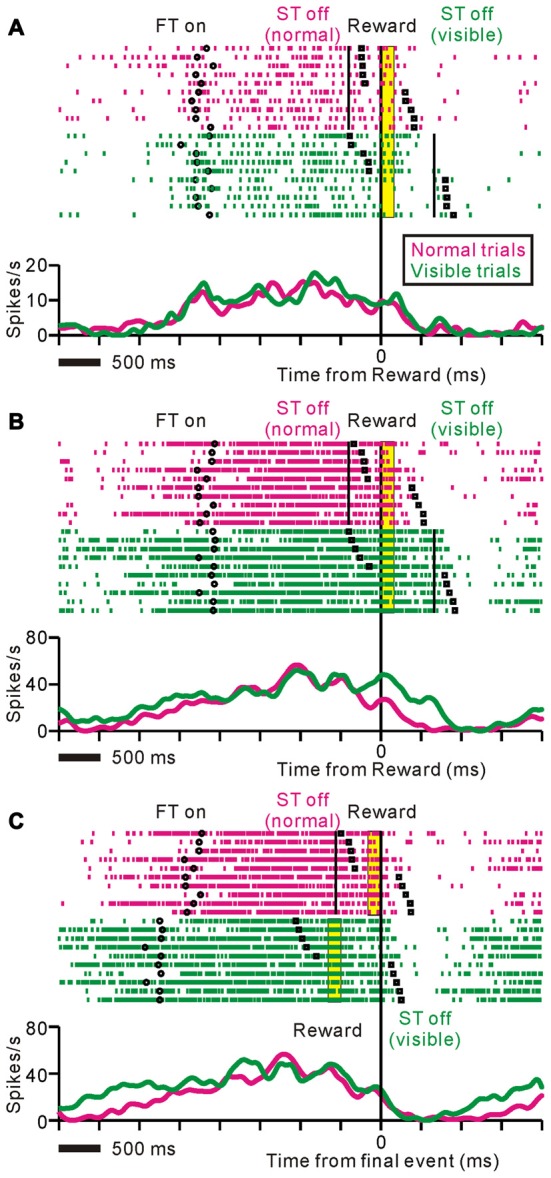
**Examples of neuronal activities during normal (magenta) and visible (green) task conditions displayed as rastergrams (*upper*) and spike density functions (*lower*).** The black circles indicate appearance of the fixation target (FT), black solid lines indicate disappearance of the ST, black squares indicate fixation break, and yellow bars indicate reward delivery period. The trials are sorted by fixation break timing relative to reward delivery. **(A)** This representative neuron showed almost identical responses during the normal and visible task conditions. The data are aligned to the time of reward delivery. **(B,C)** Examples of neuronal activity during the same set of trials are shown. The data are aligned to the time of reward delivery **(B)** and the end of an individual trial **(C)**. In the visible condition, tonic responses were sustained until the disappearance of the ST after the reward delivery.

Figure 4 shows examples of neuronal activities recorded during the normal and visible task conditions. In the normal trials, the activity of the neuron in Figure 4A increased around the time of the FT appearance, remained increased during the saccade task, and terminated shortly after the reward delivery. The monkey broke fixation around the time of the reward delivery. In the visible condition, the tonic activity also terminated shortly after the reward, while the ST remained visible after the reward delivery and the monkey tended to maintain fixation even after reward delivery. The modulation profiles relative to the reward delivery were almost identical across the two conditions. Thus, the data suggest that, regardless of the task condition or fixation behavior, this neuron might send information regarding prediction of the upcoming reward until the time of reward delivery.

Figure 4B illustrates another example of neuronal activity. This neuron exhibited an anticipatory increase in activity before the FT appeared, and maintained this activity until shortly after the reward delivery in the normal task condition. On the other hand, during the visible task condition, tonic responses were sustained until the disappearance of the ST after the reward delivery. Note that even trials that the monkey broke fixation before the reward delivery, the tonic activity was maintained until the disappearance of the ST. Figure 4C shows the same set of trials as shown in Figure 4B, aligned to the end of an individual trial such that the reward delivery in the normal trials and the disappearance of the ST in the visible trials. For this neuron, the modulation profiles relative to the end of an individual trial were almost identical across the two conditions. Thus, the tonic activity of this neuron might be related to the execution and completion of task behavior.

**Figure 5:**
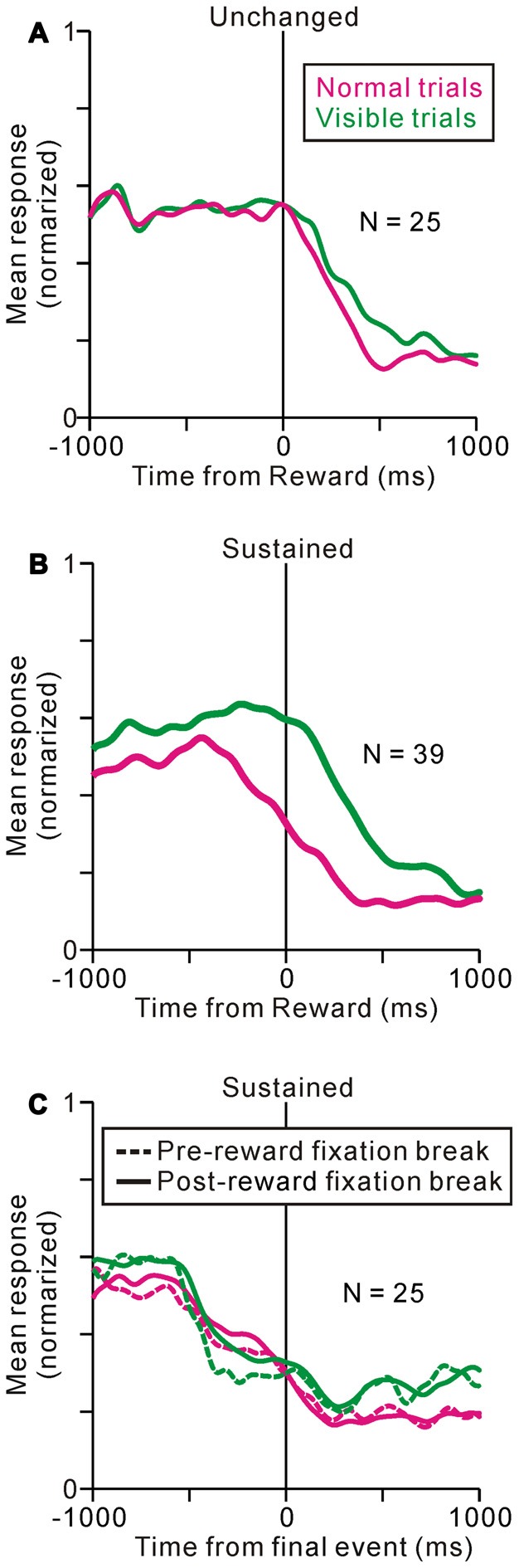
**Population average activity during normal (magenta) and visible (green) tasks are shown separately for unchanged- (A) and sustained- **(B,C)** type neurons.** The data are aligned to the time of reward delivery **(A,B)** and the end of an individual trial **(C)**. **(C)** The data are further separated into pre- (dotted line) and post-reward (solid line) fixation break trials.

Some of the neurons (37%) showed almost identical response patterns relative to the reward delivery during the normal and visible task conditions, like the example neuron in Figure 4A (unchanged-type neurons, *N* = 25, *p* > 0.05, Wilcoxon rank-sum test, Figure 5A). While most of the others (58%) exhibited sustained activity during the visible condition, and thus, were significantly more active during the visible than during the normal condition (sustained-type neurons, *N* = 39, *p* < 0.05, Wilcoxon rank-sum test, Figures 4B, 5B). These neurons showed almost identical response patterns relative to the end of an individual trial, both for pre- and post-reward fixation break trials (Figure 5C, data are shown for 25 sustained-type neurons that have sufficient data for four conditions). Thus, these tonic activity was not related to actual fixation break itself. Only a minority of neurons exhibited lower activity during the visible condition (*N* = 3). As we mentioned above, during the normal task condition, many tonic increases in activity terminated shortly after the reward delivery, while other neuronal activity terminated shortly before that (see Figure 2B). Both types of neuronal groups included unchanged- and sustained-types of activity modulations during the visible condition. Some tonic active PPTg neurons (10 unchanged- and 16 sustained-type neurons) showed saccade-related phasic changes in activity, but the saccade-direction dependent differential responses ceased before the reward period. Another subset of the tonic active PPTg neurons (4 unchanged- and 16 sustained-type neurons) showed higher activity during the task period in the visible condition than the normal condition, as seen in Figure 5B. One possible explanation is that the visible task condition requires higher motivational demands than normal task condition, and the tonic activity reflects motivation of the monkey. It is also possible that this group of sustained-type neurons simply takes longer for their activity to decay following reward delivery in visible trials. There were no significant differences in recording location (*p* = 0.3, Wilcoxon rank-sum test) or spike duration (*p* = 0.06, Wilcoxon rank-sum test) between the unchanged- and sustained-type of neurons.

**Figure 6:**
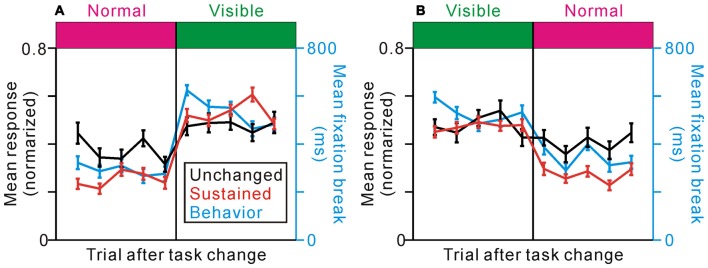
**Effects of task change on the response of unchanged- (black) and sustained- (red) type neurons and on the fixation behavior (cyan) in the change from the normal to visible (A) and the visible to normal **(B)** conditions.** The responses represent the average firing frequencies collected from 0 ms to 600 ms after the reward delivery, normalized to the peak responses of the individual neurons.

We then analyzed fixation behavior and neuronal responses during the transition phases between the two different task conditions for unchanged- and sustained-type neurons (Figures 6A,B). In the first trial after changes in task condition, monkeys could not predict the condition change because there was no apparent cue, but the fixation behavior changed immediately with actual task condition (*p* < 0.001 for the normal to visible condition and *p* = 0.048 for the visible to normal condition, Scheffé test, comparing the response in the first changed trial with the previous 5 trials). The unchanged-type neurons exhibited similar activities before and after the task condition change (*p* = 0.95 for the normal to visible and *p* = 0.99 for the visible to normal condition, Scheffé test). On the other hand, the activities of the sustained-type neurons changed after the task condition change (*p* < 0.001 for both conditions, Scheffé test), even in the first trial after the task change (*p* < 0.001 for the normal to visible and *p* = 0.029 for the visible to normal condition, Scheffé test). Thus, the activity of the sustained-type neurons changed with the actual task event and fixation behavior, which is different from the reward predicting activity of PPTg neurons (Okada et al., 2009).

**Figure 7:**
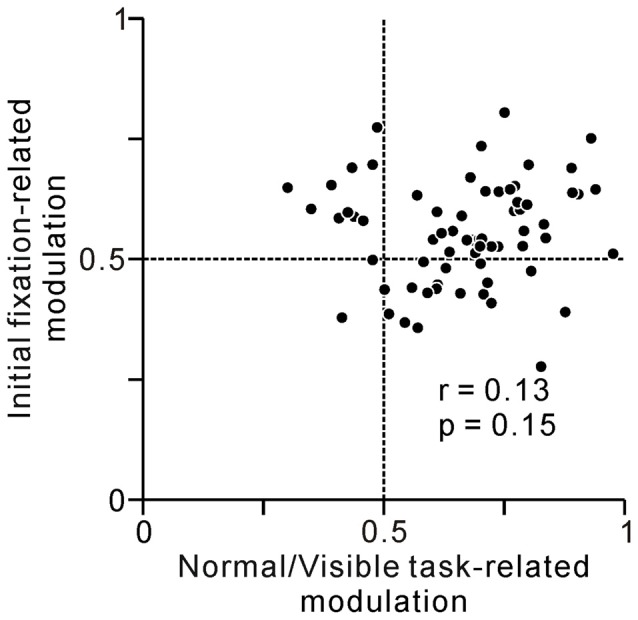
**A plot of normal/visible task-related modulation (*x*-axis) vs. initial fixation-related modulation (*y*-axis).** Task-related activity modulation at the last phase of the saccade task was measured by comparing average firing rates at the reward period between normal vs. visible trials using ROC analysis. Behavior-related modulation at the initial phase of the task was measured by comparing average firing rates at 0–600 ms before the appearance of the initial FT between short vs. long reaction times to FT trials using ROC analysis.

As we previously reported, some tonic changes in activity were related to anticipatory fixation behavior (Okada and Kobayashi, 2013). Thus, one working hypothesis is that, the activity of one group of neurons changed solely related to prediction of reward, while that of others were related solely to execution of fixation behavior. If the tonic activity of some PPTg neurons reflected fixational behavior both in the initial and last phase of the task, these neurons showed higher value both for normal/visible task- and initial fixation-related modulations. We analyzed the relationship between the normal/visible task-related modulation and initial fixation-related modulation (Figure 7), but there was only a poor correlation (*r* = 0.13, *p* = 0.15, Spearman’s rank correlation). Thus, PPTg neurons encode behavioral and reward information in a rather complex manner.

**Figure 8:**
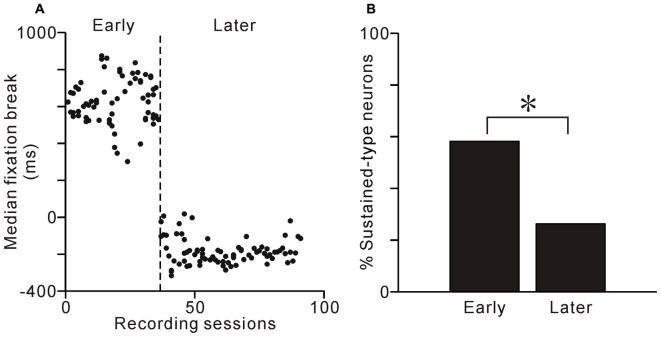
**Learning-related changes in behavior and proportions of neuron types. (A)** Median time of fixation break relative to reward delivery are plotted against recording sessions for monkey 1. **(B)** Proportion of sustained-type neurons during the early and later periods. The proportion of sustained-type neurons was smaller in the later period than in the early period. *p < 0.01, *χ*^2^ analysis.

After much training and many recording sessions, monkey 1 changed its fixation behavior and tended to break fixation before reward delivery, even during the visible task condition (Figure 8A). In this later period, the fixation-break behavior of monkey 1 was identical for the two task conditions (*p* = 0.99 for the normal to visible and *p* = 0.24 for the visible to normal conditions, Scheffé test). Thus, the monkey 1 might change behavioral policy and ignore the ST in visible condition. We then questioned whether, if tonic neuronal activity were related to the conditioned task event and monkey’s fixation behavior, the proportion of sustained-type neurons might decrease in the later period. During this later period, neuronal activity was recorded from 81 neurons during both normal and visible task conditions. The proportion of sustained-type neurons was indeed smaller during this period compared to the early period (Figure 8B, *χ^2^* = 9.9, df = 2, *p* < 0.01, *χ*^2^ analysis). Thus, at the population level, the tonic activity of PPTg neurons was related to task event and fixation behavior.

## Discussion

These experiments were designed to determine whether the tonic activity of PPTg neurons was related to the prediction of reward or execution of task behavior. By using a modified version of a visually guided saccade task, we could distinguish between reward epoch and the end of an individual trial in time. Nearly 40% of the PPTg neurons seem to send information about the prediction of the upcoming reward until the time of reward delivery, while the activity of nearly 60% of them seemed to be related to the execution of task behavior. These findings suggest that in addition to the reward value information, which is essential for the computation of reward prediction error in reinforcement learning, some PPTg neurons contribute to the execution of the conditioned task behavior.

### PPTg Encodes Reward Prediction Signal

The tonic activity of one group of PPTg neurons ceased shortly after reward delivery, both for the normal trials, when the monkeys had already broken their fixation, and for the visible trials, when the monkeys’ gazes remained fixed (Figures 4A, 5A). Thus, regardless of the task condition or monkey’s fixation behavior, this type of PPTg neurons might send the information regarding prediction of the upcoming reward until the time of reward delivery. The reinforcement learning theory assumes that the key information necessary to associate action and outcome is the reward prediction error signal that is implemented in dopaminergic neurons (Schultz, 2002; Bromberg-Martin et al., 2010). The PPTg is one of the excitatory input sources to dopaminergic neurons (Mena-Segovia et al., 2008; Watabe-Uchida et al., 2012; Dautan et al., 2016). The tonic activity of PPTg neurons that was maintained until reward delivery regardless of the monkeys’ behavior, matches the requirement of the reward prediction signal that is necessary to compute the reward prediction error in dopaminergic neurons.

Lesioning the PPTg impaired probabilistic reversal learning by reducing the sensitivity to positive reward feedback (Syed et al., 2016), and specifically, after inactivation of the posterior PPTg, rats showed no sign of omission learning (MacLaren et al., 2013). Moreover, lesioning the PPTg attenuated lever pressing for *d*-amphetamine in rats, but if the rats had already learned the task before the lesion, some of the deficits were ameliorated (Alderson et al., 2004). The results of these studies suggest that PPTg neurons might play a role in learning, especially in acquiring new action-outcome associations. Moreover, recent studies reported that selective lesions of cholinergic PPTg neurons did not impact responding and learning (Steidl et al., 2014; MacLaren et al., 2016), suggesting that non-cholinergic PPTg neurons are responsible for reward processing and learning. However, in our current experiments, it was difficult to determine the neurotransmitter of the recorded PPTg neurons (Boucetta et al., 2014); further studies are needed to identify the relationship between the neurochemical identity and response property of these neurons.

### PPTg Contributes to the Execution of Conditioned Task Behavior

The tonic activity of another group of PPTg neurons sustained until the end of an individual trial. In the visible task, the ST remained visible and the monkeys tended to maintain fixation even after reward delivery, and in these cases, some neuronal activity was also sustained after reward delivery compared to that during the normal task condition (Figures 4B, 5B). Furthermore, the tonic activity sustained until the end of an individual trial, both for early and later fixation break trials (Figures 4C, 5C). These changes in activity occurred even in the first trial after task change, implying that it was related to the actual task event and fixation behavior and not to the prediction of upcoming events (Figure 6B). Additionally, after several recording sessions when monkey 1 began to perform the normal and visible tasks similarly, the proportion of the sustained-type neurons was less (Figure 8B). These observations suggest that this type of PPTg neuron contributes to the execution of conditioned task behavior, which is consistent with a similar view of Gut and Winn (2016). Classically, the PPTg was regarded as a locomotor center (Garcia-Rill and Skinner, 1988) because stimulation of the PPTg area induced locomotion (Garcia-Rill et al., 1987). However, recent studies reported that lesioning the PPTg did not disrupt locomotion, but affected conditioned task behavior (Inglis et al., 1994; Condé et al., 1998; MacLaren et al., 2014). Another line of studies viewed the PPTg as part of the ascending reticular activating system that maintains the motivational and attentional state of animals (Steriade, 1996). Lesioning the PPTg did produce deficits in sustained attention (Kozak et al., 2005), especially selective lesions of cholinergic neurons (Cyr et al., 2015). The behavior-related tonic activity of PPTg neurons might maintain the motivational and/or attentional state of the monkey and contribute to the successful completion of task behavior.

### Involvement of the PPTg in Parkinson’s Disease

Recently, the PPTg was highlighted by its relation to Parkinson’s disease (PD), a neurodegenerative disorder and whose main pathophysiology is a loss of dopaminergic neurons, but the neuronal loss was also reported in the PPTg in PD patients (Pahapill and Lozano, 2000). Recent imaging studies revealed that, in PD patients with freezing of gate, fractional anisotropy was reduced and mean diffusivity values were increased in the PPTg (Youn et al., 2015) and structural connectivity with the PPTg was also altered (Schweder et al., 2010; Fling et al., 2013). Freezing of gait is not a simple motor symptom, but rather a deficit in initiation and execution of movement (Shine et al., 2011). PD patients with freezing of gate also exhibit disturbances in upper limb movement (Nieuwboer et al., 2009), voluntary saccades (Walton et al., 2015), and speech (Park et al., 2014). The findings of a recent study suggested roles of the PPTg in the initial analysis of sensory data and in rapid decision making (Gut and Winn, 2016). Moreover, PD patients with freezing of gate showed no pre-cue effect, whereas healthy controls exhibited faster reaction times in response to loud auditory stimuli (Thevathasan et al., 2011). The freezing of gait and postural instability in PD are resistant to dopaminergic medication (Bloem et al., 2004), and deep brain stimulation of the PPTg has emerged as an effective treatment for these symptoms (Ferraye et al., 2010; Moro et al., 2010). Deep brain stimulation of the PPTg might restore dopaminergic and non-dopaminergic function in PD patients and contribute to the execution of conditioned behavior.

## Author Contributions

KO and YK: conceived and designed the experiments, performed the experiments, analyzed the data and wrote the article.

## Conflict of Interest Statement

The authors declare that the research was conducted in the absence of any commercial or financial relationships that could be construed as a potential conflict of interest.
